# Highly Promiscuous Oxidases Discovered in the Bovine Rumen Microbiome

**DOI:** 10.3389/fmicb.2018.00861

**Published:** 2018-05-04

**Authors:** Lisa Ufarté, Gabrielle Potocki-Veronese, Davide Cecchini, Alexandra S. Tauzin, Angeline Rizzo, Diego P. Morgavi, Bernard Cathala, Céline Moreau, Megane Cleret, Patrick Robe, Christophe Klopp, Elisabeth Laville

**Affiliations:** ^1^Laboratoire d’Ingénierie des Systèmes Biologiques et des Procédés, Centre National de la Recherche Scientifique, Institut National de la Recherche Agronomique, Institut National des Sciences Appliquées de Toulouse, Université de Toulouse, Toulouse, France; ^2^INRA, UMR 1213 Herbivores, Saint-Genès Champanelle, France; ^3^UR1268 Biopolymères Interactions Assemblages, INRA, Nantes, France; ^4^LibraGen S.A, Toulouse, France; ^5^Plateforme Bio-informatique Toulouse Genopole, UBIA INRA, BP 52627, Castanet-Tolosan, France

**Keywords:** bovine rumen, microbiome, functional metagenomics, lignin degradation, redox enzymes, biorefining, dye bioremediation

## Abstract

The bovine rumen hosts a diverse microbiota, which is highly specialized in the degradation of lignocellulose. Ruminal bacteria, in particular, are well equipped to deconstruct plant cell wall polysaccharides. Nevertheless, their potential role in the breakdown of the lignin network has never been investigated. In this study, we used functional metagenomics to identify bacterial redox enzymes acting on polyaromatic compounds. A new methodology was developed to explore the potential of uncultured microbes to degrade lignin derivatives, namely kraft lignin and lignosulfonate. From a fosmid library covering 0.7 Gb of metagenomic DNA, three hit clones were identified, producing enzymes able to oxidize a wide variety of polyaromatic compounds without the need for the addition of copper, manganese, or mediators. These promiscuous redox enzymes could thus be of potential interest both in plant biomass refining and dye remediation. The enzymes were derived from uncultured Clostridia, and belong to complex gene clusters involving proteins of different functional types, including hemicellulases, which likely work in synergy to produce substrate degradation.

## Introduction

Oxidoreductases are a large family of enzymes – including laccases, alcohol oxidases, monooxygenases, mono- and oligosaccharide oxidases, lignin peroxidases, cytochrome C oxidases, NADPH oxidases, and monoamine oxidases – that catalyze oxidation–reduction (redox) reactions, and which have a broad variety of substrate specificities and reaction mechanisms. Several oxidoreductase enzymes are already being used in industrial applications, such as dye decolorization, soil and water bioremediation, and biorefining. For decades, most of our understanding of aromatic compound-degrading microorganisms has come from functional genomics or studying model microbial communities (Pieper et al., 2004). However, most of the microorganisms capable of breaking down aromatic compounds remain uncharacterized as a result of our inability to isolate and culture them. This is why the search for novelty remains a challenge. Functional metagenomics is a highly efficient tool in the search for novel biocatalysts among the huge diversity of uncultured microbes. Several metagenomic studies performed on microbial communities derived from polluted environments have led to the identification of new oxygenases involved in the degradation of aromatic compounds, which could have potential applications for bioremediation (for a review, see Ufarté et al., 2015). In 2005, Ferrer et al. (2005) discovered a polyphenol oxidase with laccase activity derived from a bovine rumen metagenome, which was the first functionally characterized member of this new enzyme family (Beloqui et al., 2006). While this study proved that redox enzymes acting on polyaromatic compounds can be retrieved from uncultured ruminal bacteria, this particular enzyme and its homologs have never been tested on lignin or its derivatives. In fact, examples of ecosystems that have been screened in order to identify enzymes involved in the degradation of lignin or lignin-derived products are scarce. A bacterial laccase acting on guaiacol, a product of lignin combustion, was first discovered while conducting activity-based screening of a metagenome sampled from mangrove soil (Ye et al., 2010). In addition, a pseudo-laccase requiring the use of exogenous Cu(II) for oxidase activity was retrieved from a coal bed metagenome being screened for lignin catabolic activity (Strachan et al., 2014). Recently, a laccase isolated from acidic bog peat using a PCR-based method was characterized and showed specificity for phenolic substrates that could be linked to lignin degradation (Ausec et al., 2017). These pioneering studies highlight the promiscuity of many oxidoreductases toward this kind of substrate, and the metagenome flexibility in loci encoding polyaromatic degrading pathways. However, the main obstacle to their discovery and characterization is the lack of experimental screens of redox enzymes, which would allow the exploration of a sufficiently large sequence space to permit such rare enzymes to be identified. This is especially true for those enzymes acting in anaerobic or microaerobic conditions, whose potential for discovery using activity-based approaches is limited (Brown and Chang, 2014). It is worth noting that such enzymes are probably very rare in certain ecosystems. For example, no laccase sequence could be found in the cow rumen metagenome studied by Ausec et al. (2011).

In rumens, lignin is present in the form of dietary plant cell wall constituents and is known to be partly degraded by anaerobic fungi and bacteria (Ruiz-Dueñas and Martínez, 2009; Abrão et al., 2017; Baba et al., 2017). One of the reasons for this could be that lignin degradation is an oxidative process, which has been described mostly with reference to aerobic ecosystems where di-oxygen can act as an electron acceptor. Due to their relative low abundance, lignin-degrading bacteria remain hard to detect and further research is thus needed to deepen our understanding of the different lignin degradation mechanisms that occur in the bovine gastrointestinal tract.

As part of this research, we used functional metagenomics to identify novel redox enzymes from uncultured ruminal bacteria. We describe a new strategy comprising three main steps that combines standard redox reactions with two innovative methods to extend the characterization of hit clones. Primary screening was performed on model substrates for redox reactions, allowing the retrieval of three isolated hit clones. Thereafter, the hit clones were used in a newly developed screening method based on colored semi-reflective films to determine their ability to depolymerize lignin derivatives. Their potential to eliminate industrial dyes was then investigated by testing their ability to degrade a panel of aromatic dyes on solid or liquid media. Finally, the sequences of the hit clones were annotated, permitting the genes involved in the catabolism of aromatic compounds, including lignin derivatives, to be identified.

## Materials and Methods

### Chemicals

The reagents used in this study were: lignin alkali (kraft lignin), 2,2’-azino-bis (3-ethylbenzothiazoline-6-sulfonic acid) diammonium salt (ABTS), 1-hydroxybenzotriazole (1-HBT), the free radical 2,2,6,6-tetramethyl-1-piperidinyloxy (TEMPO), and 3’,5’-dimethoxy-4’-hydroxyacetophenone (acetosyringone and 3,5-dimethoxy-4-hydroxybenzaldehyde (syringaldehyde) purchased from Sigma–Aldrich (France). The dyes used were: acid fuschin (AF), amaranth (A), and tropaeolin O (TO) purchased from Fisher Scientific (France); and reactive orange 16 (RO16), malachite green (MG), cibracon brillant red 3BA (CBR3BA), reactive black 5 (RB5), and remazol brilliant blue R (RBBR) purchased from Sigma–Aldrich (France). The structure of these dyes is summarized in **Table 1**.

**Table 1:** Structures of the tested dyes (AZO = azo dye; A = anthraquinonic dye; T = triarylmethane dye), and their maximum absorbance wavelength.

(A) Remazol brilliant blue R, 595 nm 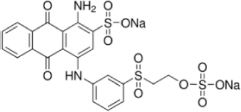	(AZO) Reactive orange 16, 385–495 nm 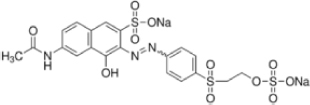

(AZO) Cibracon brilliant red 3BA, 525 nm 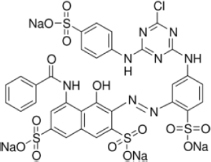	(AZO) Reactive black 5, 600 nm 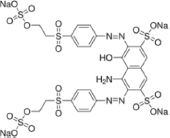

(AZO) Tropaeolin O, 390 nm 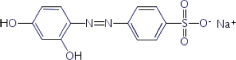	(AZO) Amaranth, 520 nm 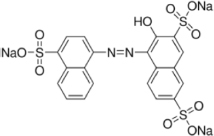

(T) Acid fuchsin, 545 nm 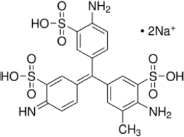	(T) Malachite green, 425–620 nm 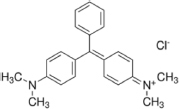

The chemicals used to produce the semi-reflective layers were: melamine-formaldehyde (MF) resin (Madurit 75%, MW 112) supplied by INEOS Melamines GmbH (Frankfurt, Germany), lignosulfonate supplied by Tembec (Montréal, Canada), xyloglucan (XG) from *Tamarindus indica* supplied by Megazyme (Bray, County Wicklow, Ireland; *M*_w_ = 202 kD; viscosity: 6.5 dL/g; sugar composition: xylose = 38%, glucose = 42%, galactose = 16%, arabinose = 4%) and poly(allylamine hydrochloride) (PAH; *M*_w_ = 120,000–200,000 g/mol) supplied by PolySciences (Germany).

### Metagenomic DNA Sampling and Library Construction

Rumen contents were obtained from two non-producing rumen-cannulated Holstein dairy cows. They were reared according to the national standards established by French legislation on animal care (Certificate of Authorization to Experiment on Living Animals, No. 004495, Ministry of Agriculture, France) at the Herbipole experimental unit (INRA, Theix). The experiments that form part of this study were approved by the Auvergne Regional Ethical committee for animal experimentation (No. 11/01438). Before sampling, the cows’ diet was altered for a period of 7 weeks to consist of 80% wheat straw, 12% concentrate, and 8% beetroot molasses. They were fed *ad libitum* once a day in the morning. Rumen contents were taken before feeding from various parts of the rumen and manually homogenized.

Enriched bacterial fractions were recovered separately from the two rumen samples which had been thawed at room temperature by applying a density gradient technique using Nycodenz such as has been described elsewhere (see Courtois et al., 2003). The cell pellets from both cows were suspended in a buffer comprised of equal parts by weight of 50 mM Tris (pH 8) and100 mM EDTA, and then incorporated into low-melt-point agarose before a gentle enzymatic lysis took place, as has also been described elsewhere (see Tasse et al., 2010). Fragments sizing between 30 and 40 kb were isolated and cloned into a pCC1FOS fosmid (Epicenter Technologies). EPI100 *Escherichia coli* cells were then transfected to obtain a library of 19,968 clones from the rumen samples. Recombinant clones were transferred to 384-well microtiter plates containing a Luria-Bertani (LB) medium, supplemented with 12.5 mg/L chloramphenicol and 8% (w/v) glycerol. They were grown for a period of 22 h at 37°C and then frozen and conserved at -80°C. All other culture media mentioned in this study contained 12.5 mg/L chloramphenicol.

### Metagenomic Library Screening

A fresh copy of the library’s 19,968 clones was gridded on 22 cm × 22 cm trays containing a solid agar medium, using an automated microplate gridder (K2, KBiosystems, Basildon, United Kingdom). A solid agar minimal medium containing 1% filter sterilized lignin alkali (w/v) as the sole carbon source was used. The minimal medium was composed of salts (3.6 g/L Na_2_HPO_4_, H_2_O; 0.62 g/L KH_2_PO_4_; 0.11 g/L NaCl; 0.42 g/L NH_4_Cl), 0.49 g/L MgSO_4_, 4.38 mg/L CaCl_2_, other salts (15 mg/L Na_2_EDTA, 2H_2_O; 4.5 mg/L ZnSO_4_, 7H_2_O; 0.3 mg/L CoCl_2_, 6H_2_O; 1 mg/L MnCl_2_, 4H_2_O; 1 mg/L H_3_BO_3_; 0.4 mg/L Na_2_MoO_4_, 2H_2_O; 3 mg/L FeSO_4_, 7H_2_O; 0.3 mg/L CuSO_4_, 5H_2_O), 0.04 g/L leucine, and 0.1 g/L thiamine hypochloride. The assay plates were incubated at 37°C for a period of between 1 and 3 weeks, depending on the time needed to visualize the growth of the hit clones.

### Functional Characterization of the Hit Clones

The hit clones isolated after primary screening due to their growth on lignin alkali as the sole carbon source were then assayed to determine the optimal conditions of ABTS oxidation and were further examined for their activity on different aromatic compounds, these being: (1) sulfonated lignin films, (2) mediators, which are model substrates used to assess oxidoreductase activity, and (3) dyes. The hit clones were grown at 37°C in 20 mL LB medium, with orbital shaking at 120 rpm. After 16 h, cells were harvested using centrifugation for 5 min at 5,000 rpm, before being re-suspended and concentrated in an activity buffer (10 mM sodium citrate buffer adjusted to optimal pHs as determined below) to obtain a final OD_600__nm_ of 80. Cell lysis was carried out using sonication. Cell debris was centrifuged at 13,000 rpm for 10 min and the cytoplasmic extracts were filtered using a 0.20 μm Minisart RC4 Syringe Filter.

Due to the potential for *E. coli* to degrade some aromatic compounds (Diaz et al., 2001; Solís et al., 2012), an *E. coli* EPI100 clone containing the pCC1FOS fosmid but without a metagenomic DNA fragment was used as a negative control.

Each experiment was carried out at least twice, and means are reported in the figures and tables included in this paper.

#### Determination of Optimal Reaction Conditions

Enzymatic reactions were carried out in 96-well microtiter plate assays using ABTS as a reductive chromogenic substrate. Each well contained 200 μL of the reaction medium: 20 μL of cytoplasmic extract, 5 mM ABTS, and 100 mM of a sodium citrate buffer at the optimal pHs. ABTS oxydation was determined by monitoring absorbance at 420 nm using a microplate spectrophotometer (BioTek^TM^ Eon^TM^ Microplate Spectrophotometers, Colmar, France). The activity of ABTS oxidation was expressed in μmol/min/L of culture, using the extinction coefficient value 𝜀ABTS (420 nm) = 36,000 M^-1^. cm^-1^.

First, we determined optimal pH at 30°C in 100 mM sodium citrate buffer supplemented with 10 mM CuSO_4_, by monitoring absorbance at 420 nm over a period of 30 min for pHs 4.0, 4.5, 5.0, 5.5, and 6.0. Then, we determined the effect of CuSO_4_ concentration at 30°C and optimal pH by monitoring absorbance at 420 nm over a period of 30 min for 0, 0.1, 1, and 10 mM CuSO_4_. Finally, optimal temperature was determined at optimal pH by monitoring absorbance at 420 nm over a period of 30 min at temperatures of 22, 30, 37, 40, 50, and 60°C.

Thermal stability was determined at the hit clones’ optimal pHs and temperatures by monitoring absorbance at 420 nm after 30 min of incubation. The optimal temperature for long-term reactions was determined at optimal pH by end-point measurement of absorbance after 17 h of reaction at 22, 30, 37, 40, 50, and 60°C. All the reactions in this study were carried out in 96-well microplates containing 200 μL of reaction medium. In order to limit evaporation, particularly for long reaction times, the microplates were covered with a lid and placed in a closed chamber saturated with moisture. The effect of H_2_O_2_ was tested at optimal pH and temperature by adding 3% (v/v) of H_2_O_2_ to the reaction medium.

#### Enzymatic Activity on Mediators

The optimal conditions for long-term reactions when using ABTS as a substrate were used to measure the activity of the three clones on different mediators. The final concentrations of substrates in the 100 mM sodium citrate buffer at the optimal pHs were 18 mM of 1-HBT and 2 mM of acetosyringone, TEMPO, and syringaldehyde; with 20 μL of cytoplasmic extract, for a total reaction volume of 200 μL. To allow substrates to be more easily compared, activity was expressed as UOD/min/L (where UOD stands for units of optical density) of culture after spectrophotometric observation at 408, 528, 300, and 370 nm, respectively. Background activity was determined under the same conditions for the biotic negative control.

#### Degradation of Semi-reflective Layers of Sulfonated Lignin

An anchoring solution of 4 g/L PAH was deposited on a silicon wafer, allowed to adsorb for 5 min, and then the support was spin-coated at 3,500 rpm for 1 min with an acceleration of 1,400 rpm/s. The MF resin mix (10% stock solution), along with lignosulfonate (100 g/L lignin stock solution, 75 g/L final) or tamarind xyloglucan (20 g/L stock solution, 10 g/L final), was deposited onto the wafer and spin-coated at 1,500 rpm for 3 min with an acceleration of 100 rpm/s. The films were put in an oven for 35 min at 135°C, then left to cool down to ambient temperature. The films were then washed in ultra-pure water for 2 h to shake off unfixed residues. Two microliters of cellular extracts was deposited on the films and incubated at the optimal temperature for 3 h in a saturated humidity atmosphere. The films were then washed with ultra-pure water and dried before observation.

#### HPAEC-PAD Analysis

The hydrolytic activity of Clone 1 on tamarind xyloglucan was examined using HPAEC-PAD. Enzymatic reactions were carried out at 37°C by adding 100 μL of xyloglucan solution (5 mg/mL final concentration) to 100 μL of cytoplasmic extract from the hit clone. After 24 h incubation, the samples were diluted by a factor of 10 with MilliQ water and analyzed using HPAEC-PAD on a Dionex ICS-3000 System (Dionex) equipped with a CarboPac PA100 column. The analyses were carried out at 30°C with a flow rate of 0.25 mL/min and the following multi-step gradient: 0–30 min (0–60% B), 30–31 min (60–0% B), and 31–36 min (0% B). The solvents used were 150 mM NaOH (eluent A) and 150 mM NaOH, and 500 mM CH3COONa (eluent B). The EPI100 *E. coli* strain harboring the empty pCC1FOS vector was used as a control. It was not able to degrade xyloglucan under the same conditions.

#### Dye Discoloration Assays

##### Selective growth on solid media with dyes as the sole carbon source

The dye decolorizing activity of clones was first screened on agar plates containing agar with a minimal medium (composition above), 1 mM CuSO_4_, and 70 ppm of dyes as the sole carbon source (excluding the agar itself) in a sodium citrate buffer (100 mM final concentration) at the optimal pHs determined for the three clones. The plates were inoculated with the hit clones as well as the *E. coli* EPI100 control, and incubated at 37°C for a period of 5 days.

##### Discoloration of liquid reaction media

The optimal conditions determined for long-term reactions were used to quantify dye discoloration in liquid media. The experiment was carried out over a period of 72 h, in 96-well microtiter plates containing a final concentration of 70 ppm of dye dissolved in 100 mM sodium citrate buffered at the optimal pHs, and 20 μL of cellular extract, for a final volume of 200 μL. The reaction media were centrifuged at 3,700 rpm for 10 min. The absorbance spectrum of each supernatant was measured using a microplate spectrophotometer (BioTek^TM^ Eon^TM^ Microplate Spectrophotometers, Colmar, France) at the initial and final reaction times. The dye discoloration yield was calculated using the decrease in absorbance at the wavelength of maximum absorbance, determined for each dye between the initial and final reaction times as follows: discoloration (%) = ([(*A*_0_-*A*_72_)/*A*_72_] - [(*A*_0_CTL_-*A*_72_CTL_)/*A*_72_]) × 100, where *A*_0_ and *A*_0_CTL_ are the initial absorbance of the dye in the cellular extract of the metagenomic clone and the *E. coli* control, respectively, at initial reaction time; and *A*_72_ and *A*_72_CTL_ are the absorbance of the dye in the cellular extract of the metagenomic clones and the *E. coli* control, respectively, after the reaction had taken place (Phugare et al., 2011). The spectra for the RO16 and MG dyes produced two _max_ values, both of which are reported in the “Results” section.

### Metagenomic Sequence Analysis

The fosmid DNA of the clone hits was extracted using the NucleoBond Xtra Midi kit from Macherey-Nagel (France) on the recommendation of the supplier. Fosmids were then sequenced using the MiSeq System (Illumina) at the GeT-PlaGe Genomics Platform (Auzeville, France). Read assembly was performed using Masurca^1^. The contigs were cleaned from the pCC1FOS vector sequence using Crossmatch^2^. The three annotated contig sequences were deposited in the DDBJ/ENA/GenBank Nucleotide Sequence Database under accession numbers LT674548, LT674550, and LT674549. Open-reading frames (ORFs) of at least 20 amino acids were predicted using MetaGene (Noguchi et al., 2006). ORF functions were inferred based on BLASTX analysis against the NCBI non-redundant and environmental databases (*e*-value < 10^-8^, identity > 35%, and query length coverage ≥ 50%). The ORFs were assigned to clusters of orthologous groups of proteins (COGs) using RPS-BLAST analysis against the COG database (*e*-values ≤ 10^-8^). A comparison was performed using BLASTP analysis against the Laccase and multicopper oxidase engineering database (LccED) (*e*-value < 10^-8^, identity ≥ 20%, and query length coverage ≥ 20%) (Sirim et al., 2011). The protein signatures were detected using the InterProScan web service^3^ (Jones et al., 2014).

Contig taxonomic assignment was carried out using MEGAN v5.10.6 (Huson et al., 2011), based on BLASTX analysis against the non-redundant NCBI database (min. score = 35, min. support = 1). Contigs were assigned to a class, genus, or species only if at least 50% of the ORFs were assigned to the same organism.

## Results and Discussion

### Metagenomic Library Screening

The library consisted of 19,968 *E. coli* fosmid clones, covering in total 0.7 Gbp of the metagenomic DNA from the rumen bacteria, with each clone containing a 30–40 kb DNA insert. The library was first screened for the ability to metabolize a depolymerized product of native lignin, namely lignin alkali, used as the sole carbon source for metagenomic clone growth. Lignin alkali, or kraft lignin, is the main by-product produced during the alkaline sulfide treatment of lignocelluloses in the pulp and paper industry. A minimal medium with kraft lignin such as this has already been used to isolate strains able to degrade this substrate (Raj et al., 2007). However, this screen has never been used to identify enzymes from metagenomic libraries, in which each clone only contains a small fraction of the genome from the native bacterium, limiting its substrate harvesting and metabolic potential. The functional assay carried out as part of this study allowed three clones to be identified that were able to grow in a mineral medium with kraft lignin as a the sole carbon source. The hit frequency was 0.015%. This is a value comparable to the hit yield found for oxidase screening in the rumen ecosystem (0.007% in Beloqui et al., 2006), although the latter was not specific to the degradation of lignin-related products. In contrast, the hit frequency was lower than that obtained from environments contaminated with polyaromatic compounds, such as activated sludge (0.09% in Suenaga et al., 2007) and oil-contaminated waters (0.2% in de Vasconcellos et al., 2010 and 3% in Silva et al., 2013).

### Characterization of Redox Activity

#### Determination of Optimal Reaction Conditions

**FIGURE 1:**
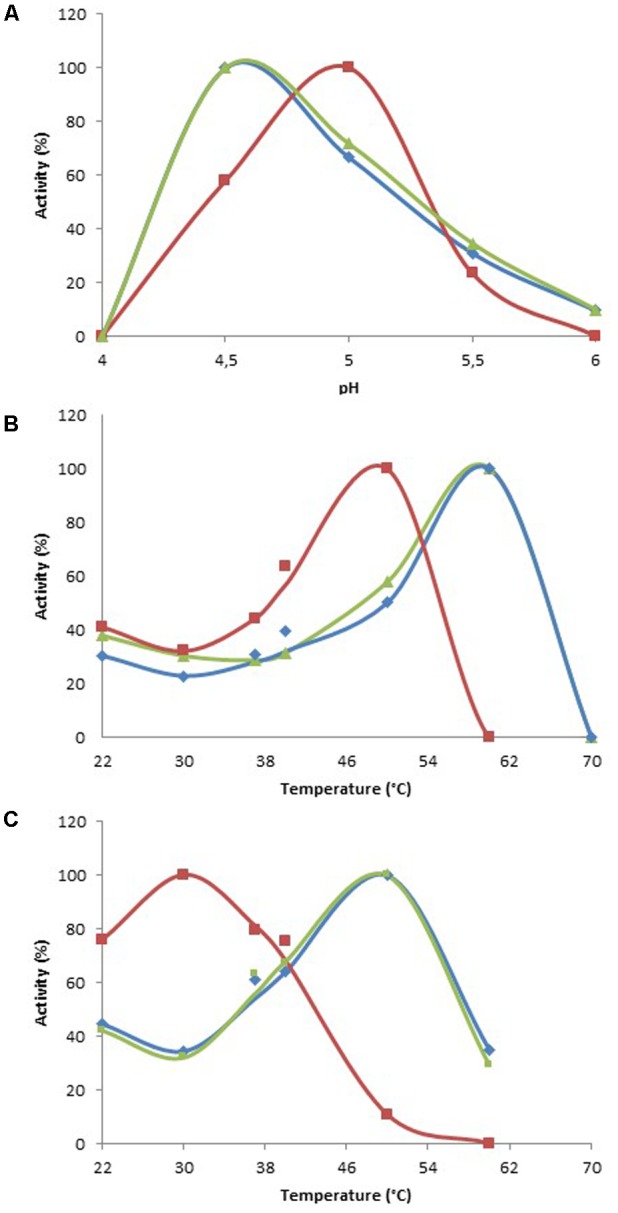
Optimal reaction conditions for ABTS oxidation. **(A)** Determination of optimal pH at 30°C. **(B)** Determination of optimal temperature at optimal pH. **(C)** Determination of optimal temperature at optimal pH, after 17 h of incubation at each temperature. Data are expressed as a percentage of the highest value for each clone. Blue, Clone 1; red, Clone 2; green, Clone 3.

The enzymatic characterization of the metagenomic clones allowed enzyme stability to be assessed, as well as the enzymes’ versatility and efficiency toward structurally different substrates, i.e., mediators and dyes. All the assays were performed using cell extracts, which contained both the recombinant enzymes, and molecules from the cellular metabolism of *E. coli* such as ions, cofactors, and even enzymes. In order to characterize the substrate specificity of the three clones, optimal conditions of activity were determined by monitoring the oxidation of ABTS at various pHs and temperatures (**Table 2**). All clones displayed optimal activity at acidic pH values, optimal pH being 4.5 for Clones 1 and 3, and 5.0 for Clone 2. Oxidative activity was totally lost when the pH value was higher than 6.0 (Clones 1 and 3), and with Clone 2 it was lost after the pH reached 5.5 (**Figure 1A**). The optimal temperature was 60°C for Clones 1 and 3, and 50°C for Clone 2 (**Figure 1B**). However, after 30 min at their optimal temperatures, only 13, 11, and 31% of activity remained for Clones 1, 2, and 3, respectively, indicating a moderate thermal stability for the enzymatic extracts. The optimal reaction temperature was thus determined according to the stability of the extracts over a 17 h incubation period. Optimal reaction temperatures were found to be 50°C for Clones 1 and 3, and 30°C for Clone 2 (**Figure 1C**).

**Table 2:** Optimal conditions for ABTS oxidation for the hit metagenomic clones.

Optimal conditions	Clone 1	Clone 2	Clone 3
pH	4.5	5.0	4.5
Temperature (°C)	60	50	60
Temperature for long-term reactions (°C)	50	30	50

The oxidative enzymes of Clones 1, 2, and 3 were not laccases or peroxidases: the addition of a copper metal or hydrogen peroxide ion to the reaction media had no significant effect on the reaction rate of the clones.

#### Enzymatic Activity on Model Substrates

Using optimal reaction conditions (**Table 2**), substrate specificity was characterized by comparing the activity of the cell extracts on five mediators whose structure and redox potential were known: ABTS, TEMPO, HBT, acetosyringone, and syringaldehyde. Syringaldehyde and acetosyringone are phenolic compounds, and are two of the main products of the degradation of syringyl-rich lignins. They are characterized by the presence of two methoxy substituents in ortho positions of the phenol, which lowers their redox potential. They have stable radicals, since the substituents have a steric hindrance effect on polymerization due to radical fusion. The TEMPO molecule is a stable radical characterized by a nitroxyl group that benefits from the steric protection provided by the four methyl groups adjacent to the nitroxyl group N–O_2_. The methyl groups prevent a double bond occurring between carbons adjacent to nitrogen. ABTS contains two sulfonate groups that can be deprotonated. The HBT substrate is characterized by an N–OH group for which enzymatic oxidation is mediated by the formation of the highly active nitroxyl radical >N–O^∙^, caused by the removal of an electron followed by the release of a proton (Camarero et al., 2005; Morozova et al., 2007; Tavares et al., 2008; Torres-Duarte et al., 2009; Pardo et al., 2013).

**FIGURE 2:**
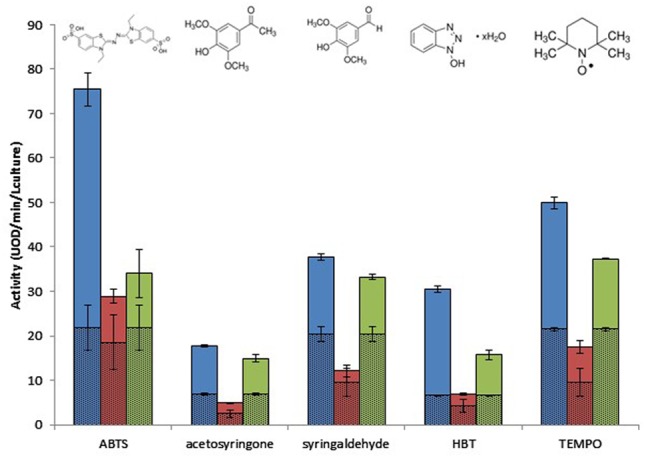
Effect of mediator type on the metagenomic clones’ oxidative activity after 17 h of incubation, at optimal pH and temperature. *Escherichia coli* activity is represented by hatched lines. Blue, Clone 1; red, Clone 2; green, Clone 3.

Clone 1 was active on all substrates and displayed the highest activity compared to the other two clones (**Figure 2**). Clone 2 displayed low levels of activity on all substrates, although activity was nevertheless detectable on all substrates, especially TEMPO. Clone 3 was the most significantly active on acetosyringone and syringaldehyde. Overall, the three clones exhibited a considerable degree of flexibility toward structurally different substrates, with Clones 1 and 3 demonstrating the highest efficiency. **Figure 2** shows the background noise for *E. coli*, confirming that it has the enzymatic machinery for oxidation. This background noise varied depending on the mediator, and also on whether Clone 2 or Clones 1 and 3 were being tested, due to differences in reaction conditions (**Table 2**).

#### Lignin-Derivative Depolymerization

**FIGURE 3:**
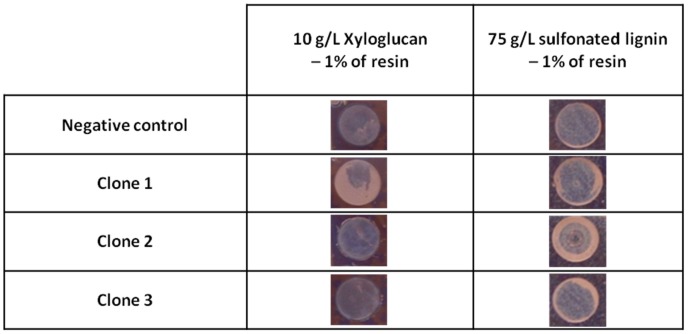
Degradation of semi-reflective layers of xyloglucan and sulfonated lignin by the hit clones.

The results of the primary screening indicated the presence of ruminal bacterial oxidoreductases, which may be involved in lignin degradation. Nevertheless, at this stage, we had not found evidence of their ability to break down polymeric lignin. We thus developed a depolymerization screening strategy, using colored semi-reflective films of polymeric sulfonated lignin (Cerclier et al., 2011), which is a by-product of the chemical pulping process (Lebo et al., 2001). As is the case with butterfly wings, the principle of this method is based on structural colors; that is, colors arising from light interference and not the presence of dyes. Modulation of the color of the semi-reflective nano-layers of polymer depends on film thickness and the refractive index of the final film. Incident light hits the air–film interface, where part of it is reflected back while the rest is transmitted into the film. The second reflection occurs at the film–substrate interface. Net reflected light intensity depends on the combination of reflected light waves from both interfaces. This principle can be exploited in order to detect the degradation of enzymatic activities by polysaccharide (Cerclier et al., 2011). However, the construction of such semi-reflective nano-layers of soluble lignin derivatives has not until now been attempted. In this study, cell extracts from the three hit clones were tested for their ability to degrade a film composed of a mix of resin and sulfonated lignin. Since the resin used to polymerize the substrate onto the layers contained aromatic molecules that could be attacked by oxidative enzymes, a film composed of a mix of resin and xyloglycan served as a control to indicate the level of resin degradation. The results are presented in **Figure 3**. The abiotic control represented by the reaction buffer at pH 4.5 did not react with the polymer layers. But the biotic negative control (*E. coli* with an empty vector) slightly affected the color of both the sulfonated lignin/resin and the xyloglucan/resin films, with the color change suggesting a decrease in layer thickness. This may be due to a slight breaking down of the structure of the biopolymer/resin layers caused by the oxidative activity of *E. coli* previously observed on ABTS and other mediators.

The three metagenomic clones affected the color of the sulfonated lignin/resin layer to a greater extent than did the biotic negative control, with Clone 2 being the most effective. We also observed a slight alteration in the xyloglucan/resin layer caused by Clones 2 and 3 which was comparable to that observed for the control clone, suggesting that the *E. coli* enzymes had a slight degrading effect on the resin. In contrast, the xyloglucan/resin layer was considerably degraded by Clone 1, suggesting that it produces activity that is able to degrade xyloglucan. Moreover, we found that no mono- or oligosaccharide was produced by Clone 1 after incubation with xyloglucan (HPAEC-PAD data analysis not presented here). We therefore put forward that Clone 1 may be affecting the xyloglucan network by altering its cohesion, as was recently shown for lytic polysaccharide monooxygenases (Villares et al., 2017).

This new screening approach has thus allowed us to demonstrate that the enzymes produced by Clone 2 were able to depolymerize solid layers of sulfonated lignin. Once this method is optimized and automated for production in micro-plate format with stable sulfonated lignin layers of homogeneous thickness, it will allow large libraries to be screened for lignin-depolymerization activities, at a throughput of hundreds of thousands of assays per week. This should make it possible to massively increase the rate of discovery and engineering of microbial ligninases derived from cultivated and non-cultivated bacteria and fungi.

#### Applications in Dye Elimination

Since the metagenomic clones displayed highly flexible specificity toward aromatic substrates, they were tested for their ability to degrade a panel of eight polycyclic aromatic dyes of diverse structures (**Table 1**), in order to evaluate the potential of the produced enzymes for industrial dye elimination. Five AZO dyes, one anthraquinonic, and two triphenylmethane were tested. They all contained aromatic rings with the potential for fusion, as well as various types of functional groups linked to these aromatic rings (-OH, -CH_3_, -NCH_3_, -SO_3_H, -SO_3_Na, and -NH_2_), which have been found to promote dye mineralization (Spadaro et al., 1992).

**FIGURE 4:**
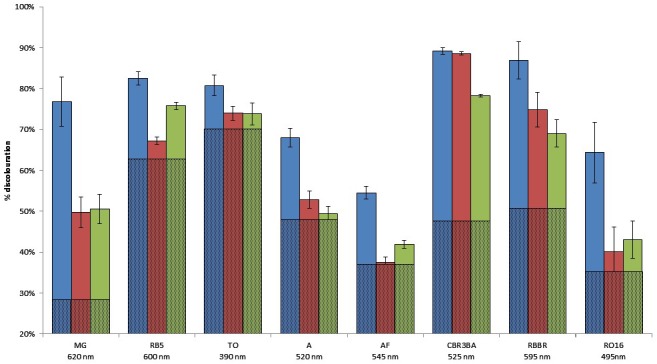
Dye degradation by the hit clones, in solid and liquid media. Histogram: quantification of the extent of liquid medium discoloration after reaction. The proportion of discoloration due to *E. coli* enzymes is represented by hatched lines. Blue, Clone 1; red, Clone 2; green, Clone 3.

**FIGURE 5:**
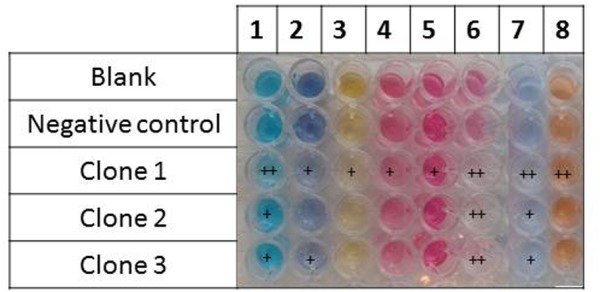
Dye discoloration caused by the hit metagenomic clones. The picture shows the supernatants of the reaction media after reaction with the cellular extracts. 1, malachite green; 2, reactive black 5; 3, tropaeolin O; 4, amaranth; 5, acid fuchsin; 6, cibracon brillant red 3BA; 7, remazol brilliant blue R; and 8, reactive orange 16.

Two types of assays were performed, these being an assessment of the ability of the metagenomic clones to metabolize the targeted dyes (by monitoring their growth on a minimal medium with the dye as the sole carbon source), and dye discoloration assays in a liquid medium. Only Clone 3 was able to grow on the selective medium with the least structurally complex dye molecule on our dye list – TO – as the sole carbon source. Cytoplasmic extracts of the three clones were then incubated in liquid media containing the dyes. After reaction, the formation of solid precipitates at the bottom of wells, associated with a discoloration of the supernatant, was observed, and this was even true to a certain extent for wells containing the control clone (**Figures 4**, **5**). Clone 1 was able to discolor all the dye solutions, while Clones 2 and 3 had a more specific effect on MG/CBR3BA/RBBR and MG/RB5/CBR3BA/RBBR, respectively. Quantification of discoloration is frequently performed in order to assess biocatalysts for dye bioremediation. Several studies have shown that aromatic compounds were either degraded or precipitated by peroxidases and polyphenol oxidases. Precipitation was attributed to the formation of phenoxy radicals followed by their spontaneous polymerization (Cooper and Nicell, 1996; Durán and Esposito, 2000; Mielgo et al., 2001; Mohan et al., 2005). Khan and Husain (2007), meanwhile, examined discoloration produced by potato and brinjal polyphenol oxidases. Dye treatment resulted in the formation of insoluble precipitates that the authors attributed to the formation of quinone-derivatives, which mediate the aggregation of aromatic pollutants. These precipitates can be easily removed from the reaction mixture by simple centrifugation, sedimentation, or filtration. Laccases also decolorize AZO dyes due to a nonspecific free radical mechanism which causes phenolic compounds to form. Their relatively low substrate specificity is associated with the use of intermediate substrates (i.e., chemical mediators) which assist in the oxidation of different substrates by facilitating electron transfer from O_2_ to the laccase substrate (Forootanfar et al., 2012; Si et al., 2013). They have the advantage of not requiring H_2_O_2_ for an oxidation reaction to be produced, as this is an expensive co-substrate (and a potential pollutant) which is considered to be responsible for dye precipitation, possibly due to free-radical formation followed by polymerization of various aromatic compounds (Bhunia et al., 2001). Few studies involving laccases have mentioned such by-products, although a study by Zille et al. (2005) did report the production due to polymerization of large numbers of coupled products, leading to a darkening of the solution. Despite the potential benefits they could bring to such depollution processes, laccases present obstacles to the biorefining of plant lignocelluloses, since they are inhibited by copper chelation caused by lignin, and also due to their double ability to depolymerize and repolymerize lignin, blocking access by cellulose- and hemicellulose-degrading enzymes to their substrates (Ruiz-Dueñas and Martínez, 2009).

Under our assay conditions, the enzymes identified as part of this study needed neither a mediator, nor the addition of copper or H_2_O_2_, in order to be active in the culture medium. This is thus of particular interest in terms of their potential industrial applications, both in biorefining and bioremediation.

### Sequence Analysis

In order to identify the genes encoding the proteins responsible for oxidative activity, the three metagenomic DNA inserts were sequenced. The reads from Clones 1, 2, and 3 were assembled into single contigs of 39.3, 22.5, and 33.7 kb, respectively. The high sequencing depth (100×) allowed accurate gene prediction. The number of predicted genes was 28, 18, and 29 for Clones 1, 2, and 3, respectively.

#### Functional Annotation

The results of the BLASTP comparison with the NCBI_NR and Swissprot protein databases, as well as protein signature detection using InterProscan, are provided in Supplementary Table S1. Mining our metagenomic sequences for laccase encoding genes by comparing these with the LccED database did not produce significant results. This would be consistent with the fact that copper is not essential to the activity we detected for these three clones. Functional annotation allowed the identification of at least one gene for each clone that might be responsible for the activity detected (Supplementary Table S1).

In Contig 1, ORF 15, annotated as D-3-phosphoglycerate dehydrogenase (PGDH), was the most probable target. The best BLASTP hit with proven activity was a distant D-lactate dehydrogenase from *Lactobacillus pentosus* (from the phylum Firmicutes; Taguchi and Ohta, 1991), which shares 29% identity across 65% of the sequence length. The results from InterProScan confirmed this annotation, highlighting a D-isomer-specific 2-hydroxyacid dehydrogenase catalytic domain, a NAD-binding domain, and an ACT C-terminal domain that is known to be related to a wide range of metabolic enzymes regulated by amino acid concentration. Other studies have already reported that dehydrogenases are involved in dye discoloration (Tilli et al., 2011). Additionally, some of them (cellobiose dehydrogenases) are redox enzymes that have an effect on the degradation of plant cell walls (Levasseur et al., 2013). In the metagenomic locus under consideration here, the dehydrogenase encoding gene is preceded by genes likely to constitute a functional cluster (ORFs 2–12) and which are involved in the transport, binding, and degradation of plant cell wall derived oligo- and polysaccharides. The dehydrogenase encoding gene is separated from the last CAZy encoding gene by two other ORFs coding for enzymes that could also participate in the redox process observed in this study, and which present similarities with enzymes from the L-serine biosynthesis pathway previously described in Dey et al. (2005). ORF 14, annotated as phosphoserine aminotransferase, and ORF 13, which possesses a methyltransferase tsaA-like domain, might indeed be involved in oxidative processes brought about by the removal of methyl groups. Indeed, like dehydrogenases, it has been shown that methyltransferases are involved in some of the degradation of lignin derivatives by fungi and bacteria (Jeffers et al., 1997; Masai et al., 2007).

In Contig 2, a cluster of three genes (ORFs 15, 16, and 17 annotated to code for a methionine synthase, a methyltransferase, and a metallo-dependent hydrolase) was proposed to code for the proteins responsible for the redox activity. ORFs 15 and 17 did not present any significant similarity with biochemically characterized proteins. The BLAST hit for the ORF 16 product which was the best characterized was the cobalamin-dependent methionine synthase (MetH) from *Thermotoga maritima*, with 66% query coverage, 36% identity, and an *e*-value of 2*e-*92. B12-dependent MetH is a large modular enzyme that uses the cobalamin cofactor as a methyl donor or acceptor in methyl transfer reactions (Evans et al., 2004). This enzyme was proposed to catalyze the conversion of 5-methyltetrahydrofolate and L-homocysteine to tetrahydrofolate and L-methionine in the final step of *de novo* methionine biosynthesis. It requires methylcobalamin as a cofactor. In addition, ORF 17 presents an amidohydrolase domain. Amidohydrolases are known to be involved in a variety of *trans*-methylations and rearrangement reactions for the transport and metabolism of amino acids, particularly methionine, where cobalamin and methyl-cobalamin are used as cofactors (Rodionov, 2003). It thus seems that the gene cluster evidenced in Clone 2 could be a new pathway of L-methionine biosynthesis. The redox activities we observed could be due to the fact that L-methionine is an essential amino acid required for a large number of important cellular functions, including the methylation of aromatic compounds by methyltransferases (Jeffers et al., 1997; Rodionov, 2004; Masai et al., 2007).

The Contig 3 sequence is particularly rich in gene coding for oxidizing enzymes. The contig contains a gene cluster organized as previously described in a study on the catabolism of phenolic compounds by facultative anaerobe bacteria (Carmona and Díaz, 2005). In these bacteria, the degradation of the phenolic substrate is caused by its reduction to a non-aromatic compound by a heterotetrameric reductase. In Contig 3, ORFs 6, 7, 8, and 9 were annotated as the δαβγ subunits of a 2-oxoglutarate ferredoxin oxidoreductase. The BLASTP hits which were best characterized were the subunits of a distant 2-oxoisovalerate oxidoreductase functionally associated with amino acid catabolism (Heider et al., 1996; Tersteegen et al., 1997). The domains predicted using InterProScan assigned the δ- and β-subunits to 4Fe-4S ferredoxin iron–sulfur and thiamine-diphosphate-binding domains, respectively. The α- and γ-subunits carried signatures of the catalytic domains of a pyruvate flavodoxin/ferredoxin oxidoreductase and a pyruvate/ketoisovalerate oxidoreductase, respectively. This complex acts on the aldehyde or oxo group of donors with ferredoxin as an acceptor and coenzyme A. It has been previously proposed that the reaction that produces reduced ferredoxin also produces a reduction in aromatic rings (Boll et al., 2002; Dorner and Boll, 2002), which would explain the phenotypes observed in this study. In addition, in Contig 3, the oxidoreductase-encoding genes were close to a gene encoding a putative transporter (ORF 2) specific to a redox cofactor, i.e., riboflavin. Other genes encoding redox proteins were found on the opposite strand of the contig. They belong to a cluster of nine genes (ORFs 11–19) of which six (ORFs 14–19) are annotated as the RnfBAEDC complex. Biegel et al. (2011) have published an extensive review of structurally equivalent complexes. These are membrane-bound electron/ion transport systems occasionally associated with cytochrome C, as would also seem to be the case with this cluster (ORFs 11 and 12), and have been found to be involved in the respiratory chain. The Rnf complex/cytochrome C association was previously shown to be co-expressed in *Methanosarcina acetivorans* (Li et al., 2006). These complexes are present in a wide variety of prokaryotes and are functionally assigned as NADH-oxidoreductase. The Rfn couples the flow of electrons from the reduced ferredoxin to NAD+ thereby generating a sodium ion gradient across the cytoplasmic membrane. In the *Acetobacterium woodii* strain, the Rnf complex is involved in the reduction of caffeate, *p*-coumarate, and ferulate, three widespread components of soil deriving from the degradation of lignin (Müller et al., 2008). Consequently, the phenotypes found in this study are the product of the involvement of multiprotein complexes that could not have been retrieved from short metagenomic DNA fragments. In addition, these multi-enzymatic systems ensure cascades of redox reactions, which most likely also involve *E. coli* cellular metabolites.

#### Taxonomic Assignment and Sequence Prevalence in the Bovine Rumen Microbiome

It was impossible to accurately assign the three metagenomic inserts from a taxonomical point of view, as their sequences were too distant from any available sequenced genome. A MEGAN analysis using low stringent criteria revealed that the sequences were probably derived from Firmicute bacteria. Indeed, 6/28, 9/18, and 5/29 ORF sequences from Clones 1, 2, and 3, respectively, were assigned to the phylum Firmicutes. It should be noted that the vast majority of ORFs from Contigs 1 and 3 (22 and 23 ORFs, respectively) were assigned to bacteria from environmental samples without any further taxonomic information other than the fact that they showed high identity to segments of two fosmid sequences from the rumen metagenome of two Jersey cows in a study by Wang et al. (2013) which involved activity-based screening of polysaccharide degradation. Contig 1 had 59% sequence coverage and 90% identity with Contig 33 from the study by Wang et al. (2013), while Contig 3 from our study showed 60% sequence coverage and 87% identity with Contig 1549a. Contigs 33 and 1549a were assigned to the phylum Firmicutes. They were retrieved from metagenomic clones active on plant cell wall polysaccharides They contained CAZy-encoding genes similar to the GH10- and GH94-encoding genes found in our Contigs 1 (ORFs 11 and 12) and 3 (ORF 28). It is interesting to note that such gene clusters were retrieved both in the course of the screening of polysaccharide degrading enzymes (Wang et al., 2013) and, in the present study, redox enzymes acting on lignin derivatives. This constitutes the first piece of evidence that bacterial putative ligninases and CAZymes can be encoded by the same genomic loci, dedicated to the degradation of walls of plant cells.

## Conclusion

In this study, functional metagenomics was used to discover new redox enzymes and metabolic pathways from the bovine rumen microbiome, active on various aromatic substrates derived from textile dyeing and the chemical treatment of lignocelluloses in the pulp and paper industries. None of these enzymes require the addition of metals or mediators to the reaction media, giving them a particular advantage over the laccases and peroxidases that are the main enzymes currently used in biorefining and dye bioremediation. Sequence analysis revealed that each of the contigs contained several redox enzymes of different functional and structural families, which probably work in synergy to degrade and metabolize the targeted substrates. In Contigs 1 and 2, the target enzymes were related to amino acid metabolism. Sequence analysis also suggested that the redox enzymes produced by the clones and identified in this study would not require the supply of oxygen. Their electron acceptors/donors would instead be, for instance, ions, cobalamin, or NAD(P), which are synthetized or absorbed in the host strain cytoplasm. Microbial processes using recombinant bacteria able to produce the entire pathways discovered here would thus be more appropriate than enzyme-based processes, which would require the addition of cofactors. In addition, two of the genomic loci discovered in this study harbor genes that encode both redox enzymes capable of acting on lignin derivatives and hemicellulases, suggesting that these bacterial enzymes could act synergistically to break down the plant cell wall network. Nevertheless, in order to accurately identify the functions of the different enzymes encoded on these loci, transcriptomic analysis and rational truncation of the fosmid inserts will be required, as well as an in-depth structural characterization of the products released from a simple model substrate. Finally, their suitability for use in biorefineries will have to be established by testing these clones on native lignin matrixes, and the way they function on this kind of substrate will also need to be studied further.

The datasets generated in the course of this study are available in the repository of the DDBJ/EMBL/GenBank Nucleotide Sequence Database under accession numbers LT674548, LT674549, LT674550^4^.

## Author Contributions

LU: acquisition, analysis, interpretation of data, and drafting the work. GP-V: conception, design of the work, analysis, interpretation of data, and drafting the work. DC, DM, BC, CM, PR: conception and design of the work and drafting. AST: design, realization, analysis, and drafting of HPLC experiment. AR, CK, and MC: acquisition, analysis of the data, and drafting. EL: conception, design of the work, data acquisition, analysis, interpretation of data, and drafting the work.

## Conflict of Interest Statement

The authors declare that the research was conducted in the absence of any commercial or financial relationships that could be construed as a potential conflict of interest.

## Footnotes

1 http://www.genome.umd.edu/masurca.html

2 http://bozeman.mbt.washington.edu/phredphrapconsed.html

3 http://www.ebi.ac.uk/interpro/

4 https://www.ncbi.nlm.nih.gov/genbank/
